# Mapping of dynamic quantitative trait loci for plant height in a RIL population of foxtail millet (*Setaria italica* L.)

**DOI:** 10.3389/fpls.2024.1418328

**Published:** 2024-07-24

**Authors:** Kangni Han, Zhilan Wang, Lin Shen, Xiaofen Du, Shichao Lian, Yuxin Li, Yanfang Li, Chuchu Tang, Huixia Li, Linyi Zhang, Jun Wang

**Affiliations:** ^1^ Hou Ji Laboratory in Shanxi Province, Millet Research Institute, Shanxi Agricultural University, Changzhi, China; ^2^ College of Agriculture, Shanxi Agricultural University, Taigu, China

**Keywords:** foxtail millet, plant height, BLUP, dynamic QTL mapping, RNA-Seq, candidate gene

## Abstract

Plant height (PH) is a crucial trait for strengthening lodging resistance and boosting yield in foxtail millet. To identify quantitative trait loci (QTL) and candidate genes associated with PH, we first developed a genetic map using a recombinant inbred line (RIL) population derived from a cross between Aininghuang and Jingu 21. Then, PH phenotyping data and four variations of best linear unbiased prediction (BLUP) were collected from nine environments and three development stages. Next, QTL mapping was conducted using both unconditional and conditional QTL methods. Subsequently, candidate genes were predicted via transcriptome analysis of parental samples at three developmental stages. The results revealed that the genetic map, based on re-sequencing, consisted of 4,360 bin markers spanning 1,016.06 cM with an average genetic distance of 0.23 cM. A total of 19 unconditional QTL, accounting for 5.23%–35.36% of the phenotypic variation explained (PVE), which included 7 major and 4 stable QTL, were identified. Meanwhile, 13 conditional QTL, explaining 5.88%–40.35% of PVE, including 5 major and 3 stable QTL, were discovered. Furthermore, four consistent and stable QTL were identified. Finally, eight candidate genes were predicted through RNA-seq and weighted gene co-expression network analysis (WGCNA). Those findings provide a crucial foundation for understanding the genetic mechanisms underlying PH development and facilitate molecular marker-assisted breeding of ideal plant types in foxtail millet.

## 1 Introduction

Foxtail millet (*Setaria italica* L., 2n=2x=18), with a cultivation history extending over 16,000 years in China (Doust et al., 2009), is considered an important source of food and fodder globally, particularly in the arid and semi-arid regions such as India and China (Singh and Prasad, 2020). Because of its higher resistance to drought, higher adaptability to infertile soils, and higher water use efficiency, foxtail millet not only is of immense significance for the advancement of ecological agriculture (Singh et al., 2024), but also plays a key role in adjusting cropping structures as a strategic reserve crop (Diao, 2019; Ramesh et al., 2023). However, increasing yield and environmental adaptability in foxtail millet, compared to other major cereal crops like maize, remains a significant potential, by developing short-statured and lodging-resistant varieties (Diao et al., 2014).

Plant height (PH), a critical agronomic trait, is closely linked to plant architecture and yield. Ideal PH can improve plant lodging resistance and photosynthesis–respiration balance as well as increase stress resistance, thereby contributing to higher yields (Stubbs et al., 2023). As a quantitative trait, PH is influenced by the intricate interplay of genetic, hormonal, and environment factors. Advances in molecular biology technology have made it easier to identify quantitative trait loci (QTL) for PH through linkage and/or association analysis. So far, more than 100 QTL for PH in foxtail millet have been identified in multiple environments and widely distributed on nine chromosomes. Among those, 24 were identified repeatedly, namely, *qPH1–1, qPH1–2*, *qPH1–3*, *qPH2–1*, *qPH2–2*, *qPH2–3*, *qPH3–1*, *qPH3–2*, *qPH3–3*, *qPH4–1*, *qPH4–2*, *qPH4–3*, *qPH4–4*, *qPH5–1*, *qPH5–2*, *qPH6–1*, *qPH6–2*, *qPH6–3*, *qPH7*, *qPH8–1*, *qPH8–2*, *qPH9–1*, *qPH9–2*, and *qPH9–3* (Mauro-Herrera and Doust, 2016; Fan et al., 2017; Feldman et al., 2017; Zhang et al., 2017; He et al., 2021). Notably, *qPH5–2*, located on chromosome 5, contributed to the highest proportion of phenotypic variation explained (PVE) (61.5%) (Mauro-Herrera and Doust, 2016). To date, five QTL or genes, *SiD2*, *SiD3*, *SiDw1*, *Seita.1G242300*, and *Seita.5G404900*, which regulated PH development through gibberellin synthesis and signaling pathways, have been fine mapped and cloned in foxtail millet (Xue et al., 2016; Fan et al., 2017; He et al., 2021; Zhu et al., 2023). Notably, *SiDw1*, a GRAS family gene encoding a DELLA protein, homologous with *GAI*/*RGA* in Arabidopsis, *SLR1* in rice, *D8* in maize, and *Rht-B1b* and *Rht-D1b* in wheat, has been functionally validated (Zhao et al., 2019).

All previously mentioned QTL and genes were identified using a conventional mapping method to analyze phenotype data at maturity. However, the development of PH is a complex and dynamic process, influenced by complex genetic networks and environment factors. Numerous genes involved in PH development exhibit dynamic expression patterns across various development stages (Che et al., 2020; Fu et al., 2022; Wu et al., 2022). To fully understand the regulatory mechanisms of PH development, it is imperative to determine the temporal and spatial expression patterns of underlying genes. Consequently, it is of paramount importance to accurately identify QTL and how to regulate PH across entire development stages.

Multiple major and stage-specific QTL for PH in crops such as wheat, cotton, and rice have been identified by dynamic QTL mapping, indicating that PH is differentially expressed throughout the plant development process (Che et al., 2020; Fu et al., 2022; Wu et al., 2022). In foxtail millet, only Mauro-Herrera and Doust (2016) carried out dynamic QTL mapping for PH in a RIL population and found that the QTL *H9a*, with a PVE ranging from 7.6% to 15.5%, had a significant and consistent impact during the vegetative growth, flowering, and harvest stages under varying environmental conditions. However, research on dynamic QTL mapping for PH throughout the entire developmental process is still scarce.

In the present study, both unconditional and conditional QTL mapping methods were employed to identify QTL associated with PH. This approach will enable us to uncover QTL that are specifically expressed only under certain conditions and those stably expressed across different environment conditions and developmental stages. By further integrating of RNA-seq and weighted gene co-expression network analysis (WGCNA), we aim to identify candidate genes potentially involved in regulating PH. These findings will not only deepen our understanding of the molecular mechanisms underlying the dynamic development of PH, but also lay a foundation for the fine mapping and cloning of QTL for PH in foxtail millet.

## 2 Materials and methods

### 2.1 Plant materials

To carry out QTL mapping, we developed an F_2:7_ recombinant inbred line (RIL) population consisting of 127 lines. This population was created using the single-seed descent method in a cross between Aininghuang (female parent, derived from natural variation of Ninghuang 1) and Jingu 21 [male parent, accession number: GPD Foxtail millet (2017)140009, obtained from Co60-irradiated Jinfen 52 dry seeds]. Aininghuang is characterized by a dwarf phenotype of approximately 110 cm, whereas Jingu 21 typically attained a taller stature, approximately 180 cm. The RIL population, along with two parents, was cultivated in three distinct experiment sites in Shanxi Province (China), during three crop seasons (2020–2022). The sites included Datong (DT, 39.3°N, 113.3°E), characterized by a cold–dry climate, with growing season temperatures ranging from 15°C to 25°C, and an annual precipitation of approximately 400 mm; Jinzhong (JZ, 37.6°N, 112.7°E), characterized by a semi-arid climate where summer temperatures often exceed 30°C and annual precipitation is approximately 450 mm; and Changzhi (CZ, 36.2°N, 113.1°E), which has a humid subtropical climate with temperatures ranging from 20°C to 28°C and an annual precipitation of approximately 550 mm. A randomized complete block design (RCBD) (Federer, 1955) with three replications was used. Each experimental block comprised two parents and all 127 lines of the RIL population. A wide–narrow row planting pattern, with a wide row of 0.48 m and a narrow row of 0.18 m, was used to cultivate all plant materials. Each plot was allocated to two rows with 2 m in length. Thinning was conducted to ensure a density of 25 plants per row once the third leaf emerged.

### 2.2 Phenotype measurement and analysis

PH was dynamically evaluated at three key development stages: jointing (T_1_), heading (T_2_), and harvest (T_3_). At T_1_ and T_2_, PH measurement was taken from the soil surface to the apex of the main stem; at T_3_, PH was measured from the soil surface to the tip of the main panicle. For phenotyping, five well-grown plants from the middle of one row, randomly selected in each line, were investigated. Net increases in PH were quantified as ΔT_1–2_ and ΔT_2–3_, representing the increases from T_1_ to T_2_ and from T_2_ to T_3_, respectively. Generalized heritability (*H* ^2^) was classified into three levels: low (less than 20%), medium (between 20% and 40%), and high (more than 40%) (Wang et al., 2018).

### 2.3 Re-sequencing

Genomic DNA from leaves of the biparent and RILs was isolated via the CTAB method (Chen and Ronald, 1999). Re-sequencing was carried out according to the CASAVA 1.8 (Illumina, Inc., San Diego, CA, USA). Genomic DNA was sheared into ~350-bp fragments for library construction. Paired-end reads (150 bp) were sequenced using the HiSeq 2500 system (Illumina, Inc., San Diego, CA, USA), with the target sequencing depth for each sample set at 30×. Raw reads were filtered based on barcode sequences. After trimming, clean reads were aligned to the Yugu1 genome sequence (*Setaria italica v2.2*) using the Burrows–Wheeler Aligner (BWA) software (Li and Durbin, 2009). Duplicate marking was performed using Picard tools (Picard, http://sourceforge.net/projects/picard/), and Genome Analysis Toolkit (GATK) (McKenna et al., 2010) was used for InDel realignment and base recalibration as part of the preprocessing steps. Subsequently, GATK was utilized again for the detection and filtering of single-nucleotide polymorphisms (SNPs) to obtain the final set of SNP sites. Finally, SNPs identified between two parents were considered polymorphic, with those exhibiting an aa×bb pattern being selected for subsequent analysis.

### 2.4 SNP genotyping and genetic map construction

To enhance the quality of the genetic map, further filtration and selection of polymorphic SNP markers from the initial set of 712,243 SNPs were performed. Sliding scans across chromosomes for genotyping were determined by a window of 15 SNPs and a step size of 1 SNP. When the count of “aa” or “bb” genotypes within the window reached or surpassed 11, it was classified as “aa” or “bb”; otherwise, it was imputed and corrected to “ab” (Huang et al., 2009). In the RIL population, SNP loci were compared against parental genotypes for binning. Samples were organized based on their chromosomal positions, marking genotype transitions as recombination breakpoints and grouping corresponding SNPs into bins. Bins shorter than 10 kb and markers exhibiting significant segregation distortion were further excluded to minimize bias. Filtered bins were utilized for genotyping analysis and further segmented into various linkage groups with HighMap software (Sasaki and International Rice Genome Sequencing, P, 2005).

### 2.5 QTL mapping

PH data from the RIL population were collected from nine different environments (20CZ, 20JZ, 20DT, 21CZ, 21JZ, 21DT, 22CZ, 22JZ, and 22DT). In addition, four best linear unbiased predictions (BLUPs) were calculated to account for environmental variability. Four BLUPs were designated as follows: BLUP1 for CZ, BLUP2 for JZ, BLUP3 for DT, and BLUP4 representing a combined analysis across all nine environments. Each individual test was based on data from a single year, location, or stage, respectively. Unconditional QTL referred to the cumulative effects at T_1_, T_2_, and T_3_ stages under various environmental conditions (Fu et al., 2022; Meyer et al., 2023), while conditional QTL referred to the net genetic effects during ΔT_1–2_ and ΔT_2–3_ (Zhu, 1995; Wu et al., 1997). In total, 65 tests were conducted, consisting of 39 tests for unconditional QTL mapping and 26 tests for conditional QTL mapping. Among the unconditional QTL mapping tests, 27 were conducted at T_1_, T_2_, and T_3_ stages across nine different environments, and 12 were conducted at T_1_, T_2_, and T_3_ stages using four BLUPs. Correspondingly, in the 26 conditional QTL mapping tests, 18 were carried out during ΔT_1–2_ and ΔT_2–3_ across nine environments, and 8 were performed using four BLUPs during ΔT_1–2_ and ΔT_2–3_.

QTL mapping analysis was conducted using the Inclusive Composite Interval Mapping (ICIM) method of the IciMapping 4.2 software (Meng et al., 2015), with a stepwise distance of 1 cM and a PIN value set at 0.001. Candidate QTL were identified based on a threshold corresponding to a 0.995 confidence level through a permutation test conducted 1,000 times. QTL name was designated as “*q*+*PH*+*chromosome number*+‘-’+*number*” (Mccouch et al., 1997). Unconditional QTL and conditional QTL were distinguished by prefixes “G” and “D”, respectively. Major QTL referred to those identified at least in two tests with a LOD score greater than 3.0 and a PVE greater than 10%. Stable QTL were those identified in more than three tests (Sun et al., 2012; Fan et al., 2015).

### 2.6 RNA-seq analysis and prediction of candidate genes

RNA from two parents was prepared from the penultimate internode at T_1_ and T_2_, and the internode below the panicle at T_3_, respectively, with three biological replicates. RNA extraction and cDNA library construction were conducted by Biomarker Technologies (Beijing, China) according to standard procedures. The cDNA libraries were sequenced on the Illumina HiSeq 2500 platform with paired-end 150-bp reads. After filtering raw reads with Trimmomatic (Bolger et al., 2014), 116.45 Gb of clean reads were obtained, with the percentage of Q30 bases in each sample being not less than 92.38% (**Supplementary Table S1**). The clean reads were mapped to the Yugu 1 reference genome using Hisat2 (Kim et al., 2015), and alignments were quantified with StringTie (Pertea et al., 2015).

Gene expression level was calculated using fragments per kilobase of transcript per million fragments mapped reads (FPKM) method. Differential gene expression analysis was conducted using DESeq2 (Love et al., 2014), with criteria set for a false discovery rate (FDR) < 0.01 and a fold change ≥ 2. Following the sequencing of the library preparations on an Illumina platform and the generation of paired-end reads, a comprehensive data analysis was undertaken. This processing included quality control, comparative analysis, functional annotation of genes, SNP calling, quantification of gene expression levels, and differential expression analysis.

To elucidate the biological significance of gene expression changes, Gene Ontology (GO) and Kyoto Encyclopedia of Genes and Genomes (KEGG) pathway enrichment analyses were performed using BMKCloud (www.biocloud.net). KEGG analysis was used to find the pathways of three stages, then DEGs across three stages were identified as candidate genes for dynamic PH. Furthermore, WGCNA was conducted based on PH phenotype and differential gene expression levels to identify significantly related modules, and then the functions of the genes within these modules were then analyzed using BMKCloud.

### 2.7 Expression analysis of candidate genes by qRT-PCR

Total RNA was extracted from the penultimate internode at T_1_ and T_2_, and the internode below the panicle at T_3_, using RNAiso Plus (TaKaRa Bio Inc., Shiga, Japan), following the manufacturer’s instructions. An equal amount of 2.0 μg of total RNA was reverse-transcribed to cDNA using oligo dT primers in the PrimeScript II 1st Strand cDNA Synthesis Kit (TaKaRa Bio Inc., Shiga, Japan). Primer 3.0 (https://primer3.ut.ee/) was employed to design qRT-PCR primers based on sequences from the *S. italica v2.2* genome (https://phytozome-next.jgi.doe.gov/info/*Sitalica_v2_2* ). The qRT-PCR reaction was performed on an ABI7500 system in a 10.0-μL volume containing 1.0 μL of template cDNA, 5.0 μL of TB Green Premix Ex Tap II (TaKaRa Bio Inc., Shiga, Japan), 2.0 μL of primer (2 μmol L^−1^), and 2.0 μL of ddH_2_O. The reaction conditions were as follows: 95°C for 3 min, followed by 40 cycles of 95°C for 10 s, 58°C for 30 s, and 72°C for 30 s, followed by a melt curve at 65–95°C by increments of 0.5°C/s. The ACTIN of *S. italica* was used as the internal reference, and the relative expression levels of interested genes were calculated using the 2^−ΔΔCT^ method (Kong et al., 2019a, b). All primer sequences used in the present study are listed in **Supplementary Table S2**.

### 2.8 Statistical analysis

Descriptive statistical analysis for PH of the biparent and RIL population was conducted using IBM SPSS Statistics 17 (SPSS, Chicago, USA). Analysis of variance (ANOVA) and *H* ^2^ were processed using the QTL IciMapping 4.2 software (Meng et al., 2015; Ma et al., 2019). BLUPs were estimated using the lme4 package in R software (de Los Campos et al., 2013). The 2^−ΔΔCT^ of qRT-PCR data were calculated via Microsoft Excel 2013, and mean calculation, significance analysis, and bar graph creation were conducted using GraphPad Prism 9.5. Venn diagrams and heatmaps of differentially expressed genes (DEGs) were drawn using TBtools (Chen et al., 2023).

## 3 Results

### 3.1 PH variation

The male parent Jingu 21 consistently exhibited taller PH than the female parent Aininghuang at three stages (T_1_, T_2_, and T_3_) and during ΔT_1–2_ and ΔT_2–3_ across nine environments almost in all tests. The RIL population showed a continuous distribution across all nine environments, exhibiting relatively small skewness and kurtosis, and two-way transgressive segregation was observed almost at all stages (**Supplementary Figure S1**). Therefore, the PH of the population followed a normal distribution, making it suitable for QTL analysis (**Table 1**; **Supplementary Figure S1**).

**Table 1:** PH performance of RIL and their parents.

Environment	Stage	Parents		RIL population						
		Aininghuang	Jingu21	MAX	MIN	MEAN	SD	CV%	Skewness	Kurtosis
20CZ	T_1_	68.32	86.96	101.05	48.52	74.87	10.02	13.38	−0.36	−0.35
	T_2_	138.97	162.91	175.95	84.44	132.14	20.94	15.85	−0.58	−0.58
	T_3_	157.87	194.43	196.41	112.28	158.02	20.26	12.82	−0.53	−0.63
20JZ	T_1_	61.23	75.91	91.08	48.93	69.3	8.52	12.3	−0.16	−0.31
	T_2_	87.69	158.55	159.09	90.04	124.86	15.35	12.29	−0.09	−0.54
	T_3_	108.79	179.63	180.36	115.84	149.22	15.2	10.18	−0.03	−0.63
20DT	T_2_	82.26	152.36	150.77	82.37	123.18	15.94	12.94	−0.74	−0.15
	T_3_	99.15	180.81	198.88	108.59	154.86	18.01	11.63	−0.49	0.04
21CZ	T_1_	59.92	62.94	71.95	46	59.36	5.16	8.69	−0.13	−0.21
	T_2_	109.55	135.17	158.32	88.45	129.66	17.55	13.53	−0.98	−0.15
	T_3_	113.54	190.7	199.51	111.45	158.99	21.49	13.51	−0.48	−0.69
21JZ	T_1_	44.5	51.97	71.33	39.33	53.94	5.57	10.32	0.04	0.2
	T_2_	95.52	165.99	150.46	89.18	121.43	13.01	10.72	−0.42	−0.19
	T_3_	157.61	191.98	188.16	110.15	153.62	16.34	10.64	−0.22	−0.45
21DT	T_1_	51.87	60.93	61.34	42.37	51.65	4.13	7.99	−0.02	−0.43
	T_2_	84.74	99.05	131.82	82.87	106.75	10.48	9.81	−0.32	−0.56
	T_3_	134.31	154.88	169.61	101.72	137.55	15.54	11.29	−0.15	−0.57
22CZ	T_1_	57.67	65.04	102.87	31.83	69.12	16.08	23.27	−0.69	−0.17
	T_2_	93	149.28	185.7	95.26	139.78	18.28	13.08	−0.62	−0.17
	T_3_	110.3	186.64	194.69	116.79	161.4	19.75	12.24	−0.56	−0.67
22JZ	T_1_	55.52	125.99	98.56	24.24	69.98	14.99	21.42	−0.63	0.06
	T_2_	114.4	162.19	182.45	92.28	140.05	18.12	12.94	−0.56	−0.11
	T_3_	121.97	199.57	206.99	121.25	168.25	19.46	11.57	−0.27	−0.73
22DT	T_1_	50.92	67.56	100.96	50.83	72.13	10	13.86	0	−0.4
	T_2_	78.37	98.54	135.28	77.86	110.29	12.76	11.57	−0.57	−0.57
	T_3_	89.2	142.55	160.15	94.16	127.57	14.51	11.37	−0.19	−0.58
20CZ	ΔT_1–2_	70.65	75.95	93.65	26.79	57.37	15.08	26.28	−0.16	−0.75
	ΔT_2–3_	18.9	31.52	139.45	20.15	26.77	11.92	44.53	7.12	64.15
20JZ	ΔT_1–2_	26.46	82.64	82.77	30.27	55.66	12.34	22.16	−0.09	−0.73
	ΔT_2–3_	21.1	21.08	139.45	20.04	25.26	11.1	43.93	8.86	90.35
20DT	ΔT_2–3_	16.9	28.46	68.49	20.22	31.97	9.83	30.74	1.27	1.69
21CZ	ΔT_1–2_	35.46	90.37	93.71	31.93	70.31	14.56	20.71	−0.85	−0.1
	ΔT_2–3_	18.16	37.39	50.92	11.24	29.32	9.26	31.58	0.25	−0.88
21JZ	ΔT_1–2_	83.94	83.42	89.6	38.89	67.49	9.29	13.77	−0.51	0.45
	ΔT_2–3_	29.18	56.59	56.02	17.56	32.19	8.27	25.7	0.51	−0.14
21DT	ΔT_1–2_	53	62.3	72.71	34.92	55.1	7.74	14.05	−0.27	−0.27
	ΔT_2–3_	29.44	31.64	52.31	15.63	30.8	8.4	27.27	0.5	−0.42
22CZ	ΔT_1–2_	35.33	84.24	89.86	45.09	70.67	8	11.33	−0.43	0.45
	ΔT_2–3_	17.3	37.36	42.92	1.54	21.62	10.29	47.58	−0.03	−0.88
22JZ	ΔT_1–2_	69.85	90.92	88.03	53.35	70.07	7.67	10.95	−0.03	−0.33
	ΔT_2–3_	7.57	37.38	65.46	1.98	28.2	12.31	43.64	0.13	−0.13
22DT	ΔT_1–2_	27.45	30.99	55.02	15.46	38.16	8.28	21.69	−0.05	−0.39
	ΔT_2–3_	10.84	44.01	38.58	3.82	17.27	6.65	38.52	0.27	0.22

Almost under all environments with the exception of Jingu 21 at 22DT, the growth speed of PH in two parents and RIL population rapidly increased during ΔT_1–2_, with an average growth of 61.99 cm, and then the speed of increase slowed down during ΔT_2–3_, with an average increase of 27.34 cm (**Table 1**), indicating that the rapid growth period of PH occurred before T_2_. ANOVA revealed that there were extremely significant differences on PH in the RIL population at T_1_, T_2_, and T_3_ stages, attributing to genotype, environment, and genotype × environment interactions (*p* < 0.01). Furthermore, *H* ^2^ was 91.73%, 96.99%, and 97.81% at T_1_, T_2_, and T_3_ stages, respectively, indicating that PH exhibited high heritability within the RIL population (**Supplementary Table S1**).

### 3.2 Genetic map construction

**Figure 1:**
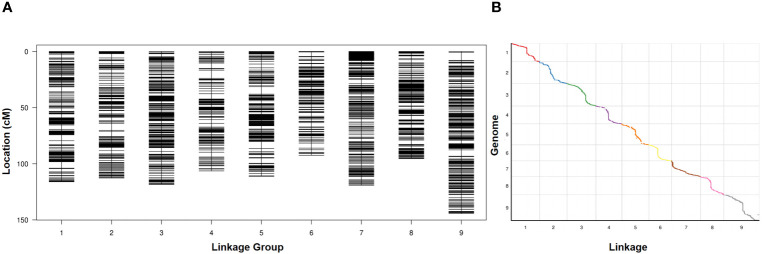
**(A)** Distribution of polymorphic markers in the genetic map constructed from the RIL population. **(B)** Collinearity analysis between the genetic map and the physical map.

RAD-seq was conducted on two parents and 127 RIL lines, and the RAD-seq data were aligned to the Yugu 1 reference genome. Aininghuang yielded 7.76 Gbp of clean reads with an average coverage of 31×, while Jingu 21 obtained 7.66 Gbp of clean reads with an average coverage of 34×. For 127 RIL lines, 88.21 Gbp of clean reads were obtained, with an average coverage of 2.97×. Finally, a genetic map was constructed using 4,360 bin markers from 712,243 SNPs, spanning 1,016.06 cM in length with an average interval of 0.23 cM between adjacent markers (**Figure 1A**). The longest linkage group, Chr. 9, spanned 144.37 cM and included 597 bin markers with an average genetic distance of 0.24 cM, whereas the shortest group, Chr. 6, spanned 92.48 cM and consisted of 300 bin markers with an average genetic distance of 0.31 cM. Collinearity analysis revealed an average Spearman coefficient up to 0.99 between the genetic and physical maps (**Figure 1B**; **Supplementary Table S3**).

### 3.3 Unconditional QTL mapping

**Figure 2:**
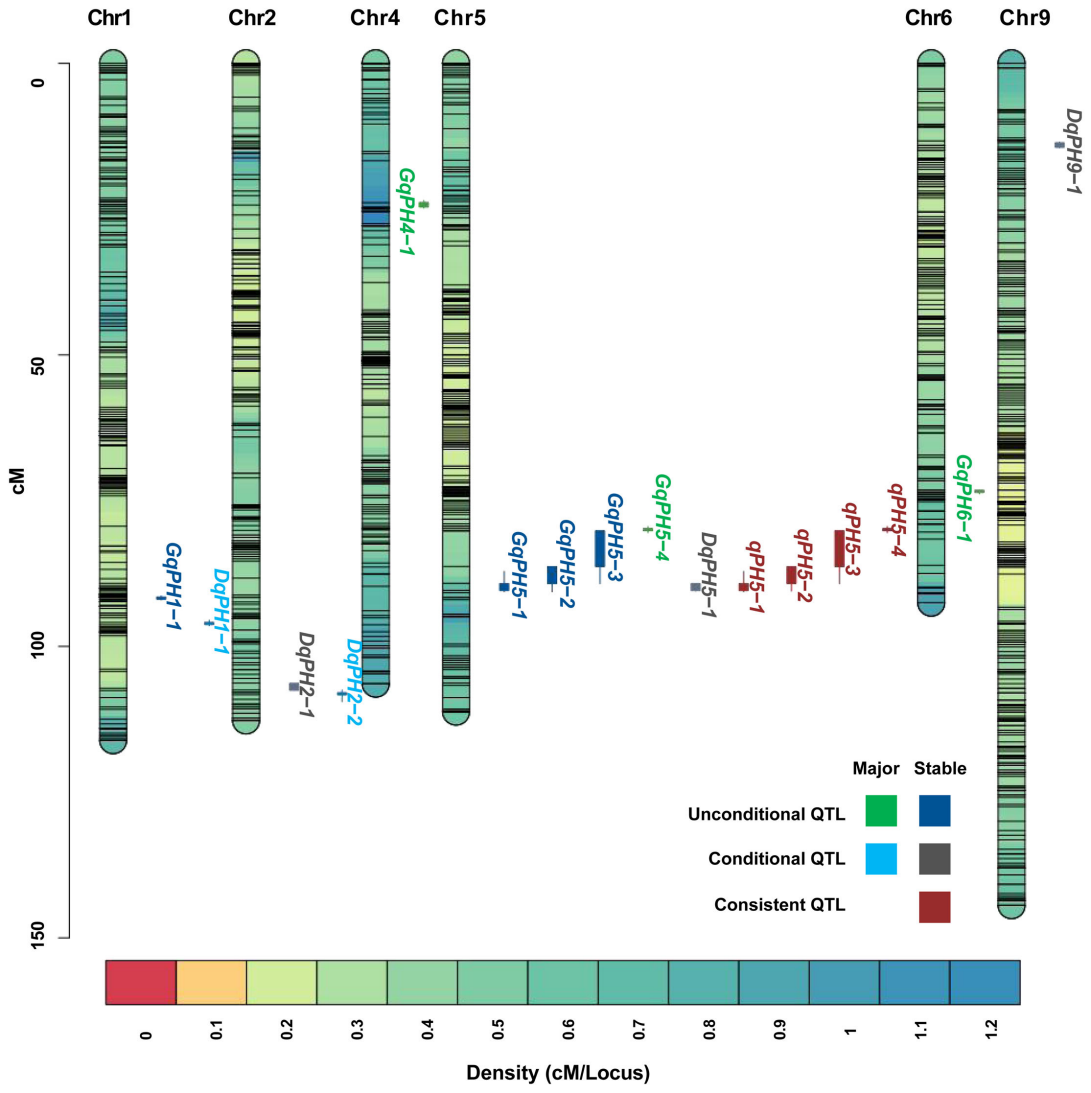
The chromosome-wise distribution of QTL for PH.

A total of 19 unconditional QTL associated with PH, including 7 major QTL, were identified at three stages under nine environments and BLUP data (**Table 2**). Among those, eight, nine, and eight QTL were identified at T_1_, T_2_, and T_3_, respectively, of which five were repeatedly identified out at least at two stages. These QTL were distributed on chromosomes 1, 4, 5, 7 and 9, respectively, characterized by PVE ranging from 5.23% to 35.36%, and LOD scores spanning from 3.02 to 16.44. Among them, 13 QTL had positive additive alleles contributed by Aininghuang, and the remaining ones were attributed to Jingu 21. Notably, four QTL, namely, *GqPH5–1*, *GqPH5–2*, *GqPH5–3*, and *GqPH1–1*, were consistently identified at least under three different conditions and were considered stable QTL (**Table 2**, **Figure 2**). Specifically, *GqPH5–2* was identified in 24 different tests, with PVE ranging from 12.67% to 34.62% and LOD scores ranging from 3.77 to 15.99. This QTL was consistently identified across two or three development stages under four BLUPs, exhibiting substantial PVE (24.91%–34.61%), which further underscores its reliability. In addition, *GqPH5–1* also exhibited notable stability across seven tests, with PVE and LOD scores ranging from 20.88% to 35.36% and 7.85 to 16.44, respectively. These results showed that PH was controlled by different QTL at various development stages.

**Table 2:** Unconditional QTL of PH identified in different environments.

QTL name	Environment	Stage	Position (cM)	Left Marker	Right Marker	LOD	PVE (%)	Additive
*GqPH1–1*	20CZ	T_2_	91.70	Block1346	Block1444	3.84	6.64	5.77
	20DT	T_2_	91.70	Block1346	Block1444	4.99	9.04	5.40
	21CZ	T_2_	91.70	Block1346	Block1444	3.02	5.78	4.56
	21CZ	T_3_	91.70	Block1346	Block1444	3.13	5.82	5.56
	21DT	T_3_	91.70	Block1346	Block1444	4.75	11.47	5.51
	22YC	T_3_	91.70	Block1346	Block1444	3.20	8.35	5.83
	22DT	T_3_	91.70	Block1346	Block1444	3.09	7.52	4.14
*GqPH1–2*	20DT	T_3_	93.80	Block1521	Block1591	3.75	9.15	5.56
*GqPH1–3*	20YC	T_3_	89.60	Block1091	Block1102	3.54	8.45	4.73
*GqPH1–4*	22YC	T_2_	93.10	Block1443	Block1560	3.95	8.48	5.62
*GqPH4–1*	22YC	T_1_	24.50	Block10555	Block10529	3.16	9.19	4.52
	BLUP2	T_1_	24.00	Block10555	Block10529	3.04	8.28	1.76
*GqPH4–2*	21DT	T_2_	58.10	Block10802	Block10805	3.02	7.97	2.92
*GqPH5–1*	20CZ	T_2_	90.30	Block13191	Block13193	16.44	35.36	13.32
		T_3_	90.30	Block13191	Block13193	13.05	30.88	11.97
	20JZ	T_2_	90.30	Block13191	Block13193	8.44	20.88	7.76
		T_3_	90.30	Block13191	Block13193	8.18	21.59	7.56
	20DT	T_3_	90.30	Block13191	Block13193	10.08	27.35	9.62
	22JZ	T_3_	90.30	Block13191	Block13193	7.85	21.90	9.45
	22DT	T3	89.60	Block13191	Block13193	9.63	26.28	7.76
	BLUP2	T_3_	90.00	Block13191	Block13193	9.70	23.67	7.98
*GqPH5–2*	20CZ	T_1_	88.20	Block13188	Block13191	15.99	34.62	6.34
	21CZ	T_1_	88.20	Block13188	Block13191	5.98	20.09	2.31
		T_2_	88.20	Block13188	Block13191	13.45	31.24	10.67
		T_3_	88.20	Block13188	Block13191	12.67	28.18	12.32
	22CZ	T_1_	88.20	Block13188	Block13191	11.04	29.02	8.86
		T_2_	87.50	Block13188	Block13191	8.48	26.53	9.74
		T_3_	88.20	Block13188	Block13191	8.02	24.91	10.33
	20JZ	T_1_	88.20	Block13188	Block13191	3.77	12.67	3.17
	21JZ	T_1_	87.50	Block13188	Block13191	6.54	21.48	2.51
	22JZ	T_1_	88.20	Block13188	Block13191	6.74	21.52	6.74
		T_2_	88.20	Block13188	Block13191	10.00	24.79	9.66
	20DT	T_2_	88.20	Block13188	Block13191	14.50	30.89	10.05
	21DT	T_3_	87.50	Block13188	Block13191	8.93	23.74	7.99
	22DT	T_2_	88.90	Block13188	Block13191	9.12	28.48	6.85
	BLUP1	T_1_	88.00	Block13188	Block13191	12.33	34.61	4.50
		T_2_	88.00	Block13188	Block13191	13.88	32.80	11.08
		T_3_	88.00	Block13188	Block13191	12.41	29.69	11.70
	BLUP2	T_1_	88.00	Block13188	Block13191	8.05	24.91	3.01
		T_2_	88.00	Block13188	Block13191	10.31	25.93	7.14
	BLUP3	T_2_	88.00	Block13188	Block13191	13.06	32.59	6.61
		T_3_	88.00	Block13188	Block13191	11.33	27.36	7.93
	BLUP4	T_1_	88.00	Block13188	Block13191	9.81	28.33	3.57
		T_2_	88.00	Block13188	Block13191	12.35	30.73	8.32
		T_3_	88.00	Block13188	Block13191	11.18	27.87	9.30
*GqPH5–3*	21JZ	T_2_	84.00	Block13150	Block13188	9.19	27.13	7.14
		T_3_	84.70	Block13150	Block13188	7.50	25.05	7.98
	21DT	T_2_	83.30	Block13150	Block13188	8.09	26.11	5.39
*GqPH5–4*	22DT	T_1_	79.80	Block13184	Block13185	7.16	22.14	4.70
	BLUP3	T_1_	80.00	Block13184	Block13185	8.48	25.01	1.86
*GqPH5–5*	21DT	T_1_	74.20	Block13071	Block13107	6.96	19.45	1.90
*GqPH5–6*	20CZ	T_3_	16.10	Block11617	Block11616	3.52	7.06	-5.81
*GqPH5–7*	20CZ	T_2_	17.50	Block11616	Block11621	3.15	5.23	-5.23
*GqPH6–1*	21CZ	T_2_	73.50	Block15848	Block15867	3.70	7.02	-5.03
	21CZ	T_3_	73.50	Block15848	Block15867	4.57	8.53	-6.74
*GqPH6–2*	BLUP1	T_1_	76.00	Block15945	Block16042	3.08	7.52	-2.09
*GqPH6–3*	BLUP1	T_2_	73.00	Block15829	Block15856	3.04	5.86	-4.65
*GqPH7–1*	21DT	T_1_	56.70	Block18649	Block18650	3.50	9.14	1.33
*GqPH7–2*	BLUP3	T_1_	54.00	Block18642	Block18645	3.19	8.63	1.11
*GqPH9–1*	20CZ	T_1_	14.70	Block23567	Block23570	4.44	7.64	-2.98

### 3.4 Conditional QTL mapping

During ΔT_1–2_ and ΔT_2–3_, eight and five QTL were identified, respectively, but no overlapping QTL were identified between them, which explained 6.5%–40.35% of PVE with LOD values ranging from 3.01 to 25.11 (**Table 3**). The positive additive effects of all QTL were contributed by Aininghuang. In total, five major QTL and three stable QTL were further identified. Notably, *DqPH9–1*, one of the three stable QTL, was a major co-localized QTL, and expressed only during ΔT_2–3_, explaining 8.04%–40.35% of the PVE under various environments. This QTL was consistently identified during ΔT_2–3_ under four BLUPs, with a substantial PVE ranging from 12.3% to 40.35%, which further underscores its reliability. Additionally, *DqPH5–1* was consistently identified during ΔT_1–2_ under four BLUPs, proving it is also a stable QTL.

**Table 3:** Conditional QTL of PH identified in different environments.

QTL Name	Environment	Stage	Position(cM)	Left Marker	Right Marker	LOD	PVE(%)	Additive
*DqPH1–1*	21DT	ΔT_2–3_	96	Block1594	Block1596	5.80	16.18	3.25
	BLUP3	ΔT_2–3_	96	Block1594	Block1596	5.99	6.50	1.56
*DqPH1–2*	21CZ	ΔT_1–2_	87	Block1084	Block1088	3.25	6.61	4.03
*DqPH1–3*	22DT	ΔT_1–2_	90	Block1092	Block1131	3.19	9.00	2.57
*DqPH2–1*	21CZ	ΔT_2–3_	107	Block5964	Block5965	4.48	10.40	2.96
	BLUP1	ΔT_2–3_	107	Block5964	Block5965	7.40	15.14	1.88
	BLUP4	ΔT_2–3_	107	Block5964	Block5965	5.63	13.08	1.64
*DqPH2–2*	22JZ	ΔT_2–3_	108	Block5970	Block5976	3.76	11.42	4.17
	BLUP2	ΔT_2–3_	108	Block5970	Block5976	4.96	14.29	1.31
*DqPH5–1*	20CZ	ΔT_1–2_	90	Block13191	Block13193	9.14	18.10	7.03
	BLUP1	ΔT_1–2_	90	Block13191	Block13193	9.11	20.74	4.48
	BLUP2	ΔT_1–2_	90	Block13191	Block13193	6.75	15.96	2.16
	BLUP3	ΔT_1–2_	90	Block13191	Block13193	9.28	22.34	1.62
	BLUP4	ΔT_1–2_	90	Block13191	Block13193	10.63	22.84	3.60
*DqPH5–2*	21CZ	ΔT_1–2_	88	Block13188	Block13191	11.09	26.90	8.19
*DqPH5–3*	21JZ	ΔT_1–2_	85	Block13150	Block13188	6.49	21.40	4.25
*DqPH5–4*	21DT	ΔT_1–2_	80	Block13184	Block13185	6.12	20.45	3.53
*DqPH5–5*	22DT	ΔT_1–2_	91	Block13197	Block13201	4.89	14.23	3.23
*DqPH9–1*	20DT	ΔT_2–3_	14	Block23566	Block23567	6.18	23.26	4.10
	21CZ	ΔT_2–3_	14	Block23566	Block23567	10.09	26.99	4.81
	22CZ	ΔT_2–3_	14	Block23566	Block23567	4.61	8.04	3.58
	22JZ	ΔT_2–3_	14	Block23566	Block23567	3.45	10.48	4.04
	22DT	ΔT_2–3_	14	Block23566	Block23567	5.46	18.77	2.86
	BLUP1	ΔT_2–3_	14	Block23566	Block23567	10.29	22.85	2.33
	BLUP2	ΔT_2–3_	14	Block23566	Block23567	4.26	12.34	1.23
	BLUP3	ΔT_2–3_	15	Block23565	Block23569	25.11	40.35	3.92
	BLUP4	ΔT_2–3_	14	Block23566	Block23567	10.46	27.09	2.38
*DqPH9–2*	20CZ	ΔT_1–2_	22	Block23638	Block23672	3.41	5.88	4.00
*DqPH9–3*	21CZ	ΔT_2–3_	78	Block26188	Block26292	3.03	6.77	2.57

### 3.5 Consistent QTL mapping

The comparative analysis between 19 unconditional and 13 conditional QTL showed that a total of 4 QTL, namely, *qPH5–1*, *qPH5–2*, *qPH5–3*, and *qPH5–4*, were consistent QTL due to their overlapping confidence intervals (**Table 4**, **Figure 2**). These QTL exhibited PVE ranging from 12.67% to 35.36% and LOD scores from 3.77 to 16.44. Notably, *qPH5–1* and *qPH5–2* were highlighted for stable expression across three experiment sites. Moreover, *qPH5–2* was identified at multiple development stages, indicating that it played a crucial role throughout the entire developmental process. In addition, *qPH5–1* exhibited stable expression across multiple environments and development stages, including that it may be a QTL with a continuous effect. However, *qPH5–4* was identified only in DT, suggesting it may possess environmental specificity or adaptability.

**Table 4:** Consistent QTL of PH identified in different environments.

QTL name	Environment	Stage	Position (cM)	Left Marker	Right Marker	LOD	PVE (%)	Additive
*qPH5–1*	20CZ	ΔT_1–2_	90	Block13191	Block13193	9.14	18.1	7.03
		T_2_	90.3	Block13191	Block13193	16.44	35.36	13.32
		T_3_	90.3	Block13191	Block13193	13.05	30.88	11.97
	20JZ	T_2_	90.3	Block13191	Block13193	8.44	20.88	7.76
		T_3_	90.3	Block13191	Block13193	8.18	21.59	7.56
	22JZ	T_3_	90.3	Block13191	Block13193	7.85	21.90	9.45
	20DT	T_3_	90.3	Block13191	Block13193	10.08	27.35	9.62
	22DT	T_3_	89.6	Block13191	Block13193	9.63	26.28	7.76
	BLUP1	ΔT_1–2_	90	Block13191	Block13193	9.11	20.74	4.48
	BLUP2	T_3_	90	Block13191	Block13193	9.7	23.67	7.98
		ΔT_1–2_	90	Block13191	Block13193	6.75	15.96	2.16
	BLUP3	ΔT_1–2_	90	Block13191	Block13193	9.28	22.34	1.62
	BLUP4	ΔT_1–2_	90	Block13191	Block13193	10.63	22.84	3.6
*qPH5–2*	20CZ	T_1_	88.2	Block13188	Block13191	15.99	34.62	6.34
	21CZ	T_1_	88.2	Block13188	Block13191	5.98	20.09	2.31
		T_2_	88.2	Block13188	Block13191	13.45	31.24	10.67
		T_3_	88.2	Block13188	Block13191	12.67	28.18	12.32
		ΔT_1–2_	88	Block13188	Block13191	11.09	26.90	8.19
	22CZ	T_1_	88.2	Block13188	Block13191	11.04	29.02	8.86
		T_2_	87.5	Block13188	Block13191	8.48	26.53	9.74
		T_3_	88.2	Block13188	Block13191	8.02	24.91	10.33
	20JZ	T_1_	88.2	Block13188	Block13191	3.77	12.67	3.17
	21JZ	T_1_	87.5	Block13188	Block13191	6.54	21.48	2.51
	22JZ	T_1_	88.2	Block13188	Block13191	6.74	21.52	6.74
		T_2_	88.2	Block13188	Block13191	10.00	24.79	9.66
	20DT	T_2_	88.2	Block13188	Block13191	14.50	30.89	10.05
	21DT	T_3_	88.9	Block13188	Block13191	9.12	28.48	6.85
	22DT	T_2_	88.9	Block13188	Block13191	9.12	28.48	6.85
	BLUP1	T_1_	88	Block13188	Block13191	12.33	34.61	4.50
		T_2_	88	Block13188	Block13191	13.88	32.80	11.08
		T_3_	88	Block13188	Block13191	12.41	29.69	11.70
	BLUP2	T_1_	88	Block13188	Block13191	8.05	24.91	3.01
		T_2_	88	Block13188	Block13191	10.31	25.93	7.14
	BLUP3	T_2_	88	Block13188	Block13191	13.06	32.59	6.61
		T_3_	88	Block13188	Block13191	11.33	27.36	7.93
	BLUP4	T_1_	88	Block13188	Block13191	9.81	28.33	3.57
		T_2_	88	Block13188	Block13191	12.35	30.73	8.32
		T_3_	88	Block13188	Block13191	11.18	27.87	9.3
*qPH5–3*	21JZ	ΔT_1–2_	85	Block13150	Block13188	6.49	21.40	4.25
		T_2_	84	Block13150	Block13188	9.19	27.13	7.14
		T_3_	84	Block13150	Block13188	7.50	25.05	7.98
	21DT	T_2_	83.3	Block13150	Block13188	8.09	26.1	5.39
*qPH5–4*	21DT	ΔT_1–2_	80	Block13184	Block13185	6.12	20.45	3.53
	22DT	T_1_	79.8	Block13184	Block13185	7.16	22.14	4.70
	BLUP3	T_1_	80	Block13184	Block13185	8.48	25	1.86

### 3.6 RNA-seq analysis

**Figure 3:**
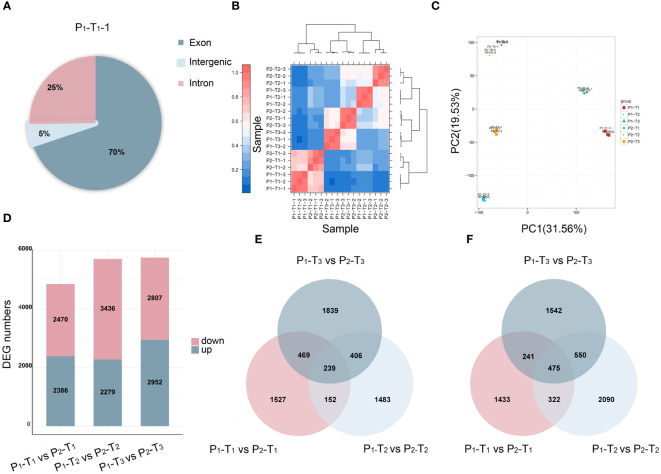
Transcriptome analysis of 18 samples. **(A)** Reads mapping. **(B)** Correlation analysis. **(C)** PCA. **(D)** DEGs at T_1_, T_2_, and T_3_ stages. **(E, F)** Venn diagrams of upregulated **(E)** and downregulated **(F)** DEGs at T_1_, T_2_, and T_3_ stages.

To further identify the key candidate genes underlying PH, we conducted a full-length transcriptome analysis. A total of 131.29 Gb of clean reads, with a GC content of 50.67%–53.00%, were obtained after removing adapter and low-quality reads. The sequencing quality was high enough (Q20 ≥ 97.81%, Q30 ≥ 92.97%) for further analysis (**Supplementary Table S4**). Upon mapping the quality-controlled sequencing data from two parents to the Yugu 1 reference genome, it was found that over 91.5% of the clean reads corresponded to the reference genome (**Supplementary Table S5**). Most of the mapped reads were concentrated in the exon region of genes (**Figure 3A**; **Supplementary Figure S2**), indicating that the sequencing results were consistent with the characteristics of RNA-Seq. Principal component analysis (PCA) revealed that two distinct groups between two parents were clustered with minimal variation among three biological replicates (**Figures 3B, C**).

A total of 4,856, 5,715, and 5,759 DEGs were identified at T_1_, T_2_, and T_3_, respectively (**Figure 3D**). Among these, 239 upregulated genes (**Figure 3E**) and 475 downregulated genes (**Figure 3F**) were overlapped and identified across T_1_, T_2_, and T_3_, respectively, suggesting that a shared group of genes played a continuous and crucial role throughout the entire process of PH development.

### 3.7 GO term and KEGG pathway enrichment analysis

**Figure 4:**
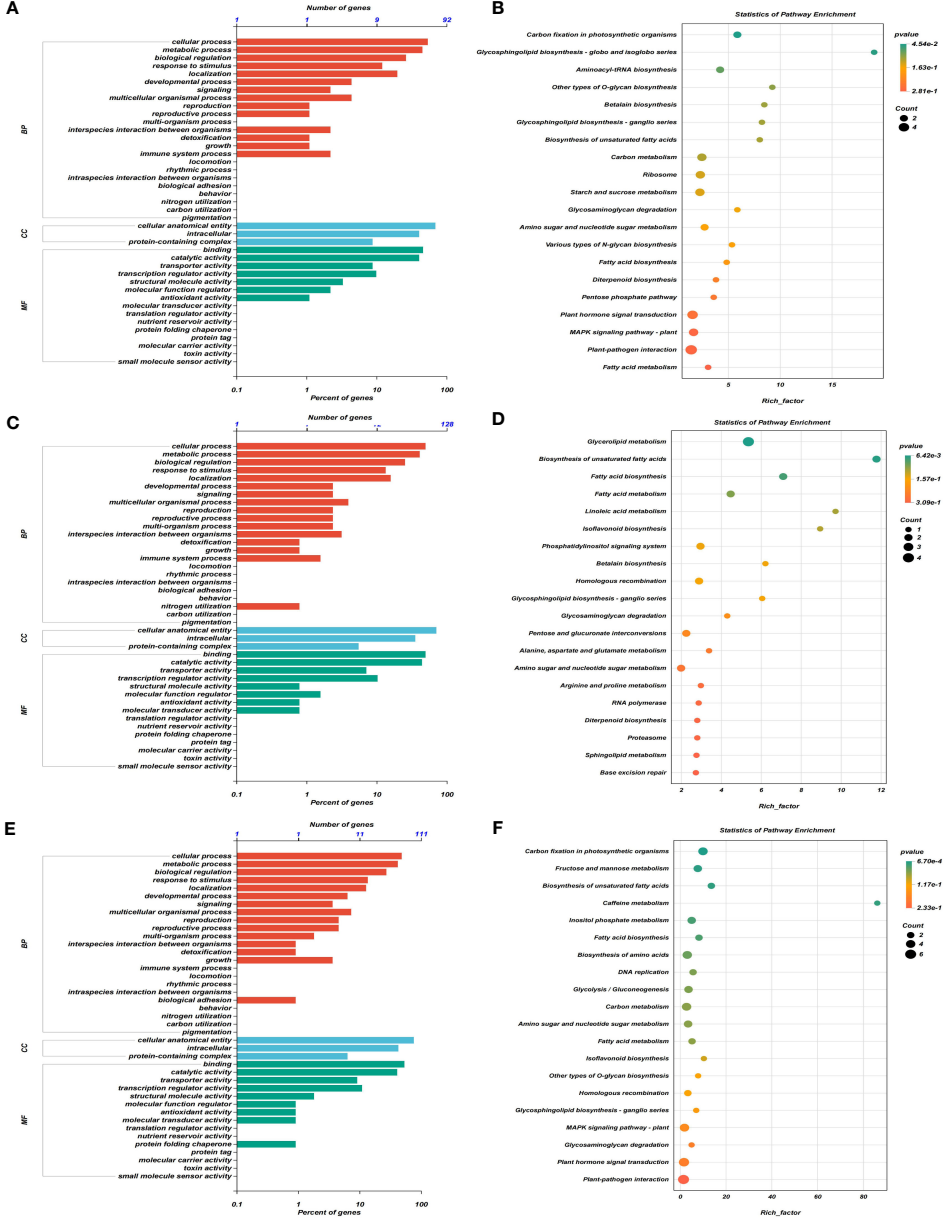
GO term and KEGG pathway enrichment analyses of DEGs obtained by RNA sequencing in four consistent stable QTL regions. **(A, C, E)** GO terms enriched for three aspects of all DEGs at T_1_ **(A)**, T_2_ **(C)**, and T_3_ **(E)** stages, respectively, in which MF represents molecular function, BP represents biological processes, and CC represents cellular components. **(B, D, F)** The top 20 terms for KEGG pathway enrichment analyses of DEGs at T_1_ **(B)**, T_2_ **(D)**, and T_3_ **(F)** stages, respectively.

GO analysis of DEGs in four consistent stable QTL regions showed that DEGs were predominantly involved in cellular processes and metabolic processes within the biological process (BP) category, cellular anatomical entity, intracellular and protein-containing complex in cellular component (CC), and binding and catalytic activity in molecular function (MF) at three stages, respectively (**Figures 4A, C, E**). Additionally, the KEGG enrichment analysis demonstrated that significant enrichment was present in carbon fixation, photosynthetic organisms and glycosphingolipid biosynthesis-globo isoglobo series at T_1_ (**Figure 4B**), glycerolipid metabolism and biosynthesis of unsaturated fatty acids at T_2_ (**Figure 4D**), and carbon fixation in photosynthetic organisms and fructose and mannose metabolism at T_3_ (**Figure 4F**).

**Figure 5:**
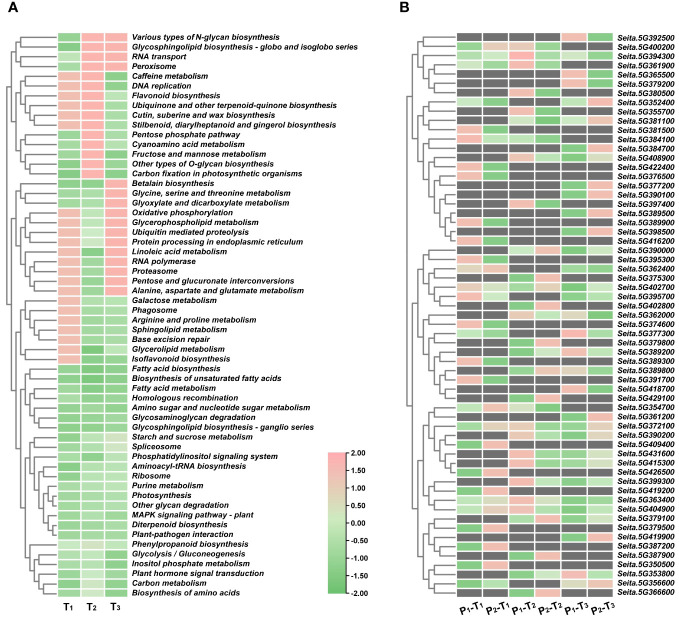
**(A)** KEGG pathway enrichment analyses of DEGs at T_1_, T_2_, and T_3_ stages in four consistent QTL regions. **(B)** Expression heatmap of 61 DEGs.

**Figure 6:**
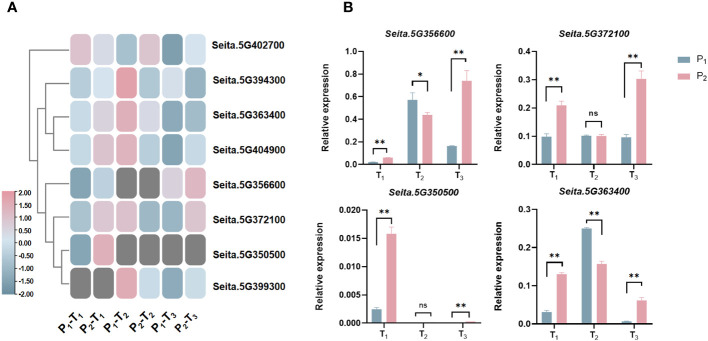
**(A)** Expression heatmap of eight candidate genes related to PH at three stages. **(B)** Expression analysis of four candidate genes in biparent via qRT-PCR at three stages. *statistically significant at *p* < 0.05; **statistically significant at *p* < 0.01; ns, not significant.

To predict candidate genes, we analyzed pathways that were significantly enriched across three stages. A total of 24 pathways were significantly enriched at T_1_, T_2_, and T_3_ stages, including fatty acid biosynthesis, biosynthesis of unsaturated fatty acids, fatty acid metabolism, homologous recombination, amino sugar and nucleotide sugar metabolism, glycosaminoglycan degradation, and glycosphingolipid biosynthesis-ganglio series (**Figure 5A**). From these pathways, we identified 61 potential candidate genes for PH within four consistent stable QTL mapping intervals. Notably, five specific genes, namely, *Seita.5G363400*, *Seita.5G372100*, *Seita.5G394300*, *Seita.5G402700*, and *Seita.5G404900*, showed significant expression differences between two parents at three stages (**Figures 5B**, **6A**).

Compared to Aininghuang, *Seita.5G372100*, *Seita.5G363400*, and *Seita.5G404900* in Jingu 21 were upregulated at T_1_, downregulated at T_2_, and upregulated again at T_3_; *Seita.5G394300* was upregulated at T_1_, then downregulated at T_2_ and T_3_. *Seita.5G402700* was downregulated at T_1_, followed by upregulation at both T_2_ and T_3_. Those expression patterns suggested that the T_1_ stage was a critical period for PH development, and these genes may be involved in key processes such as stem elongation, cell division, and hormone regulation on PH development (**Figure 6A**).

### 3.8 WGCNA

**Figure 7:**
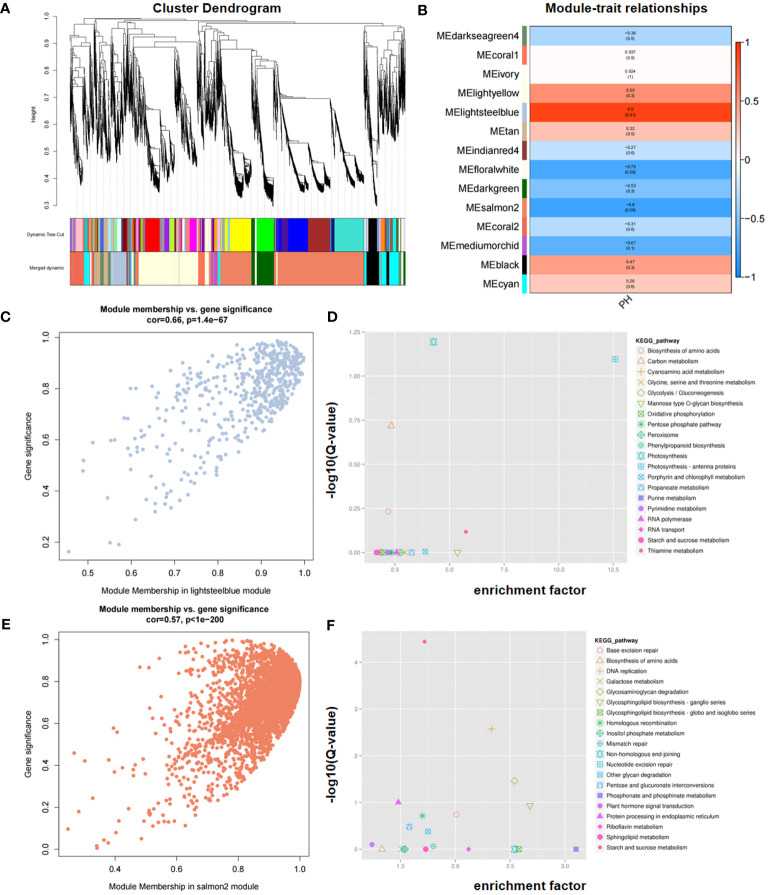
Identification of candidate genes via WGCNA. **(A)** Cluster dendrogram. **(B)** Module–trait relationships in WGCNA. **(C, E)** Correlation between MM (Module Membership) and GS (Gene Significance) in the lightsteelblue **(C)** and salmon2 **(E)** modules, respectively. **(D, F)** KEGG enrichment of genes within consistent QTL in the lightsteelblue **(D)** and salmon2 **(F)** modules, respectively. GS represents the correlation of each gene within the module, and MM represents the correlation between a single gene and its module.

To explore the gene expression network associated with PH, WGCNA was conducted using a total of 38,494 expressed genes that were detected. These genes were divided into 14 modules, of which 2, lightsteelblue and salmon2, significantly correlated with PH (**Figures 7A, B**). Scatter plot analysis revealed that there was a positive correlation between module membership (MM) and gene significance (GS) in the two modules (**Figures 7C, E**).

KEGG enrichment analysis revealed that the lightsteelblue module was significantly enriched in photosynthesis, photosynthesis-antenna proteins, carbon metabolism, biosynthesis of amino acids, and thiamine metabolism pathways, and the salmon2 module was significantly enriched in starch and sucrose metabolism, DNA replication, glycosaminoglycan degradation, protein processing in endoplasmic reticulum, and glycosphingolipid biosynthesis-ganglio series, among others (**Figures 7D, F**). Based on the KEGG enriched pathways in both modules, four genes, namely, *Seita.5G350500*, *Seita.5G356600*, *Seita.5G399300*, and *Seita.5G394300*, were identified as candidates for PH. Through the integration of KEGG enrichment analysis of DEGs in RNA-seq with WGCNA, three genes, namely, *Seita.5G350500*, *Seita.5G356600*, and *Seita.5G399300*, were consistently identified, underscoring their potential significance in modulating PH.

### 3.9 Expression analysis of candidate genes by qRT-PCR

To clarify the precise expression level of candidate genes, we randomly selected four candidates, namely, *Seita.5G350500*, *Seita.5G356600*, *Seita.5G363400*, and *Seita.5G372100*, to carry out expression analysis using qRT-PCR in the two parents (**Figure 6B**). The results demonstrated that almost all genes were differentially expressed between two parents at three development stages, which were consistent with the results of RNA-Seq (**Figure 6A**), indicating that these genes were key candidates.

## 4 Discussion

### 4.1 BLUP of quantitative trait

BLUP significantly improved predictive accuracy in both animal and plant populations (Macedo et al., 2020; VanRaden and Cole, 2020). Considering the significant influence of interactions between genes and environment on quantitative traits, phenotype data were typically collected from diverse environments to mitigate environmental impacts and enhance predictive accuracy through phenotype regression analysis of numerous genetic variants (de Los Campos et al., 2013; Ankamah-Yeboah et al., 2020). In this study, BLUP values for three experiment sites (CZ, JZ, and DT) as well as for overall were calculated. The results demonstrated a high degree of agreement between the data and the model, thereby establishing a solid foundation for QTL mapping.

### 4.2 Combination analysis of unconditional and conditional QTL mapping

Traditional QTL mapping provided information on cumulative effects at specific growth stages (Fu et al., 2022; Meyer et al., 2023). However, this approach, which focused on the final values of quantitative traits, neglected the net or incremental genetic effects of QTLs across different development processes (Zhu, 1995; Wu et al., 1997). By integrating unconditional and conditional QTL mapping, it was possible to acquire the genetic loci that affected quantitative traits at various development stages and, further, to clearly elucidate the expression patterns and effects of underlying loci throughout the developmental process (Yang et al., 2006; Cui et al., 2011; Wu et al., 2022).

In the present study, we identified eight, nine, and eight unconditional QTL at T_1_, T_2_, and T_3_ stages, and eight and five conditional QTL during ΔT_1–2_ and ΔT_2–3_, respectively, indicating selective expression at different development stages. Among those, *GqPH5–3*, *GqPH5–5*, *GqPH6–1*, *GqPH1–1*, and *DqPH9–1* overlapped with prior studies (Zhang et al., 2017; He et al., 2021), suggesting that those QTL may exhibit consistent functions or effects across different studies. Remarkably, QTL occurrence was more pronounced in the early stages of PH development, indicating that QTL expression was particularly vigorous during the initial stages, which was consistent with previous studies (Wang et al., 2019; Che et al., 2020; Wu et al., 2022).

Meanwhile, we identified four stable unconditional QTL, *GqPH5–1*, *GqPH5–2*, *GqPH5–3*, and *GqPH1–1*, and three stable conditional QTL, *DqPH2–1, DqPH5–1*, and *DqPH9–1.* Furthermore, four consistent QTL, namely, *qPH5–1*, *qPH5–2*, *qPH5–3*, and *qPH5–4*, were found to exert both net genetic and cumulative effects on PH via a combination analysis of unconditional and conditional QTL mapping. Notably, *qPH5–1* was stably expressed across three environment sites (CZ, JZ, and DT) during ΔT_1–2_ and the subsequent T_2_ and T_3_ (**Table 3**). The region of *qPH5–1* was consistent with that reported by Ni et al. (2017), indicating its stability and reliability.

### 4.3 Prediction of candidate genes via integration of transcriptome and WGCNA

Transcriptome analysis was effective in identifying genes that responded significantly to specific conditions, and WGCNA focused on identifying gene sets that may not show significant changes in expression individually but work together in biological processes (Yao et al., 2023; Xie et al., 2024). In the present study, we identified 61 DEGs associated with PH from RNA-seq, among which five genes, namely, *Seita.5G404900*, *Seita.5G363400*, *Seita.5G394300*, *Seita.5G372100*, and *Seita.5G402700*, were continuously differentially expressed throughout the development stages and considered as candidates. Furthermore, three genes, *Seita.5G350500*, *Seita.5G356600*, and *Seita.5G399300*, were identified to be differentially expressed through WGCNA. In summary, through the integration of transcriptome analysis and WGCNA, we identified eight candidate genes potentially influencing PH.

Notably, the “Green Revolution” gene *SD1* in rice, the homology of *Seita.5G404900*, has been proven to play an essential role in regulating PH (Phillips et al., 1995; Huang et al., 1998; Hedden and Phillips, 2000; Sakamoto et al., 2001; Yamaguchi, 2008; Zhu et al., 2023). Also in rice, *OFP2*, the homology of *Seita.5G363400*, decreased PH by interacting with *KNOX* and *BELL* genes to suppress gibberellin biosynthesis (Schmitz et al., 2015). *RBOHH*, the homology of *Seita.5G372100*, influenced PH by regulating reactive oxygen species (ROS) levels mediated by DELLA proteins in response to both biotic and abiotic stresses in Arabidopsis and rice (Achard et al., 2008; Zhu et al., 2024). Therefore, we randomly selected four candidates, namely, *Seita.5G350500*, *Seita.5G356600*, *Seita.5G363400*, and *Seita.5G372100*, to carry out expression analysis using qRT-PCR. The results showed that all of them exhibited differential expression between the two parents at three development stages (**Figure 6B**), which was consistent with the RNA-Seq, indicating that those candidates played a significant role in the development of PH in foxtail millet.

## 5 Conclusion

PH is a critical trait influencing lodging, stress resistance, and yield in foxtail millet. In the present study, a total of seven major unconditional QTL and five major conditional QTL for PH were identified using a high-density genetic map with 4,360 bin markers based on a RIL population, of which four QTL were simultaneously identified via unconditional and conditional QTL mapping. Within the four consensus QTL intervals, eight candidate genes were predicted through RNA-seq and WGCNA. This study laid the foundation for fine mapping and cloning of QTL for PH in foxtail millet.

## Data availability statement

The datasets presented in this study can be found in online repositories. The names of the repository/repositories and accession number(s) can be found in the article/**Supplementary Material**.

## Author contributions

KH: Conceptualization, Data curation, Formal Analysis, Funding acquisition, Investigation, Methodology, Project administration, Resources, Supervision, Validation, Visualization, Writing – original draft, Writing – review & editing. ZW: Writing – review & editing, Data curation, Methodology, Resources. LS: Data curation, Investigation, Writing – review & editing. XD: Data curation, Investigation, Methodology, Writing – review & editing. SL: Investigation, Resources, Writing – review & editing. YXL: Investigation, Writing – review & editing. YFL: Visualization, Writing – review & editing. CT: Investigation, Writing – review & editing. HL: Writing – review & editing, Formal analysis, Funding acquisition. LZ: Investigation, Resources, Writing – review & editing. JW: Conceptualization, Funding acquisition, Project administration, Resources, Supervision, Writing – review & editing.
