# Supplementary material for: Mapping of dynamic quantitative trait loci for plant height in a RIL population of foxtail millet (Setaria italica L.)
Source: Front Plant Sci. 2024 Jul 24;15:1418328. doi: 10.3389/fpls.2024.1418328 (PMC11303304; doi:10.3389/fpls.2024.1418328)
Supplement: Supplementary file 1 [file DataSheet_1.docx]

**Supplementary Figure S1** Histogram of the frequency distribution of plant height across nine environments at T_1_, T_2_ and T_3_ stages. Red line represents density; Blue line represents normal distribution curve.


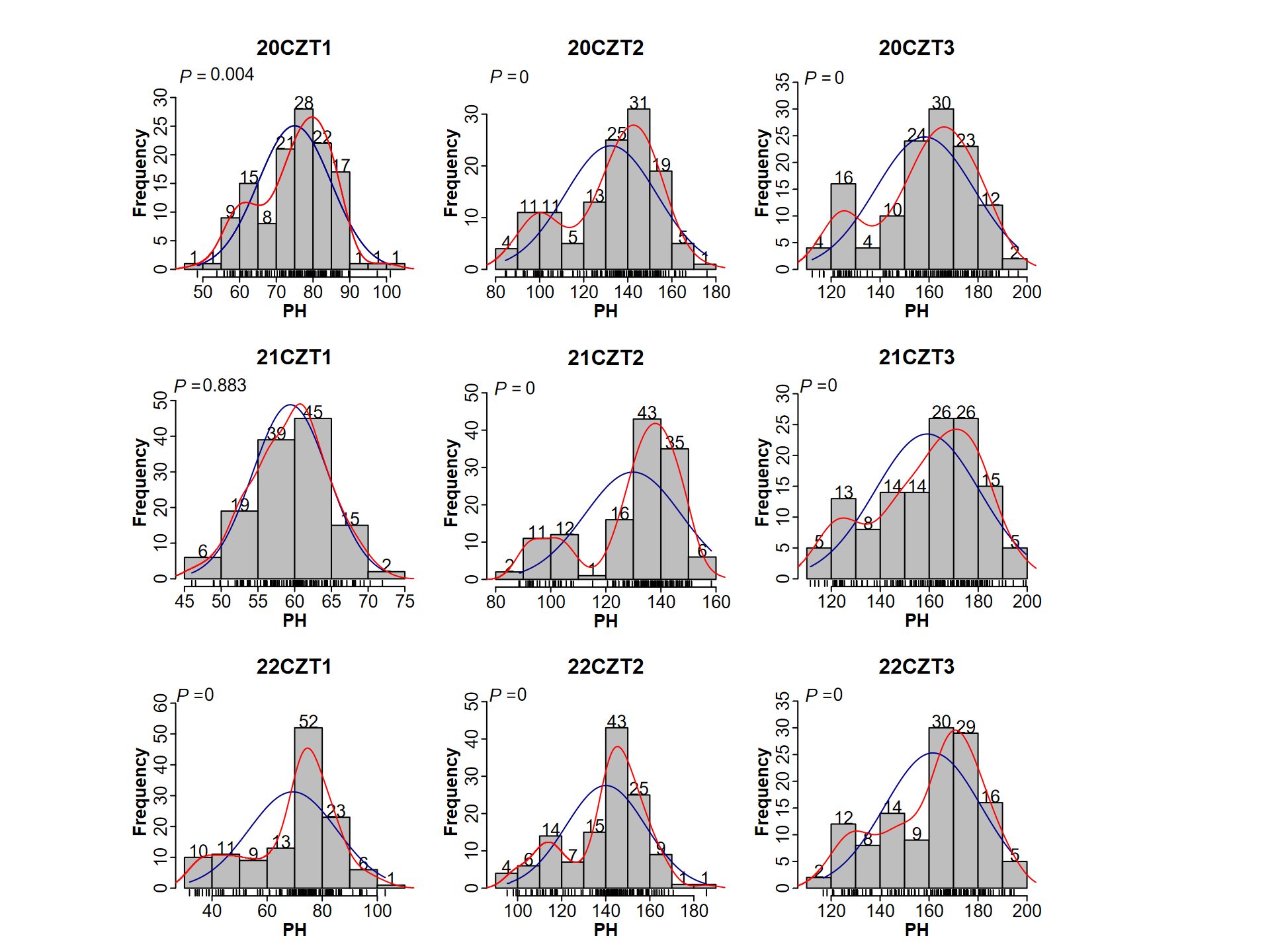

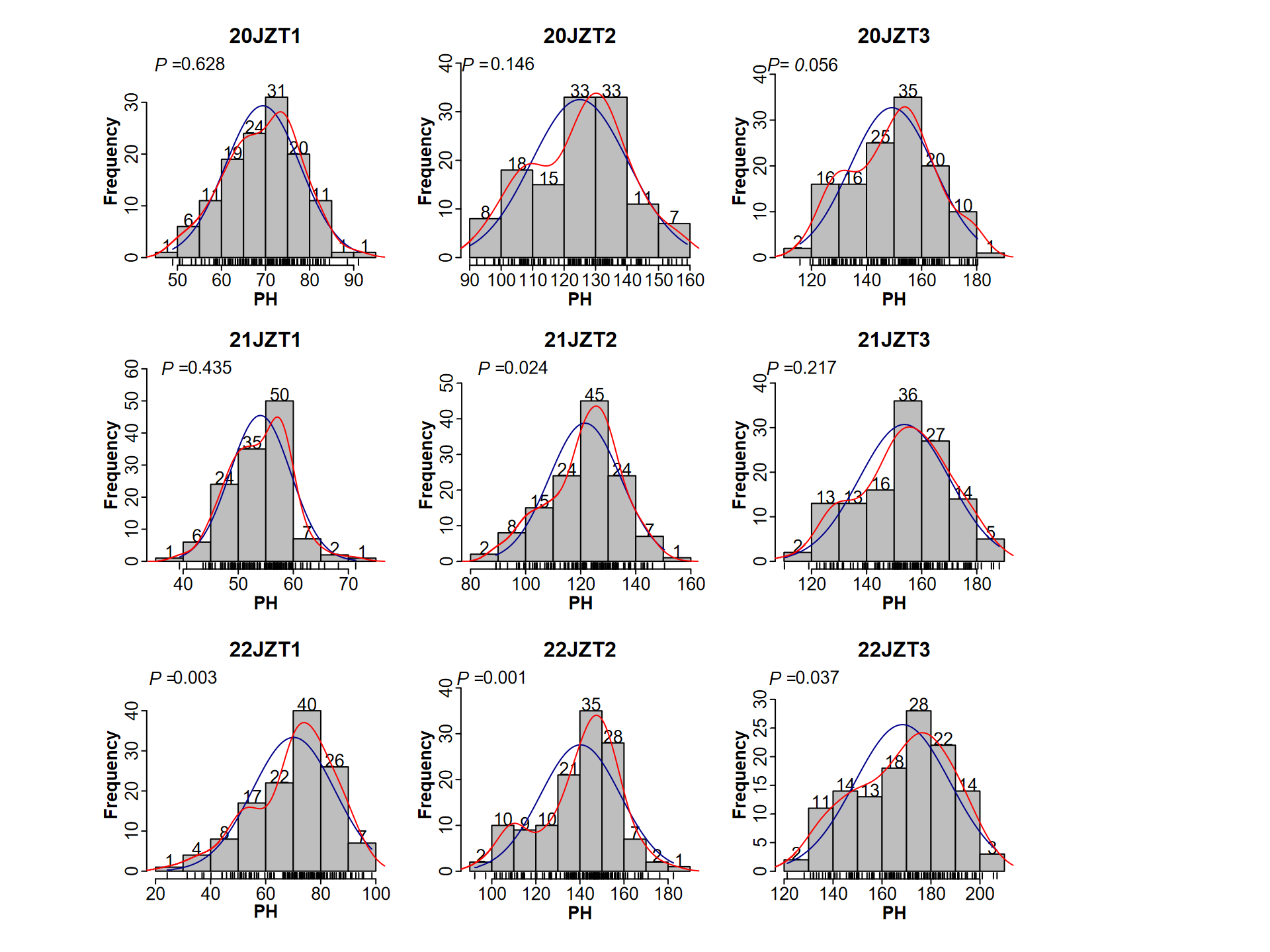

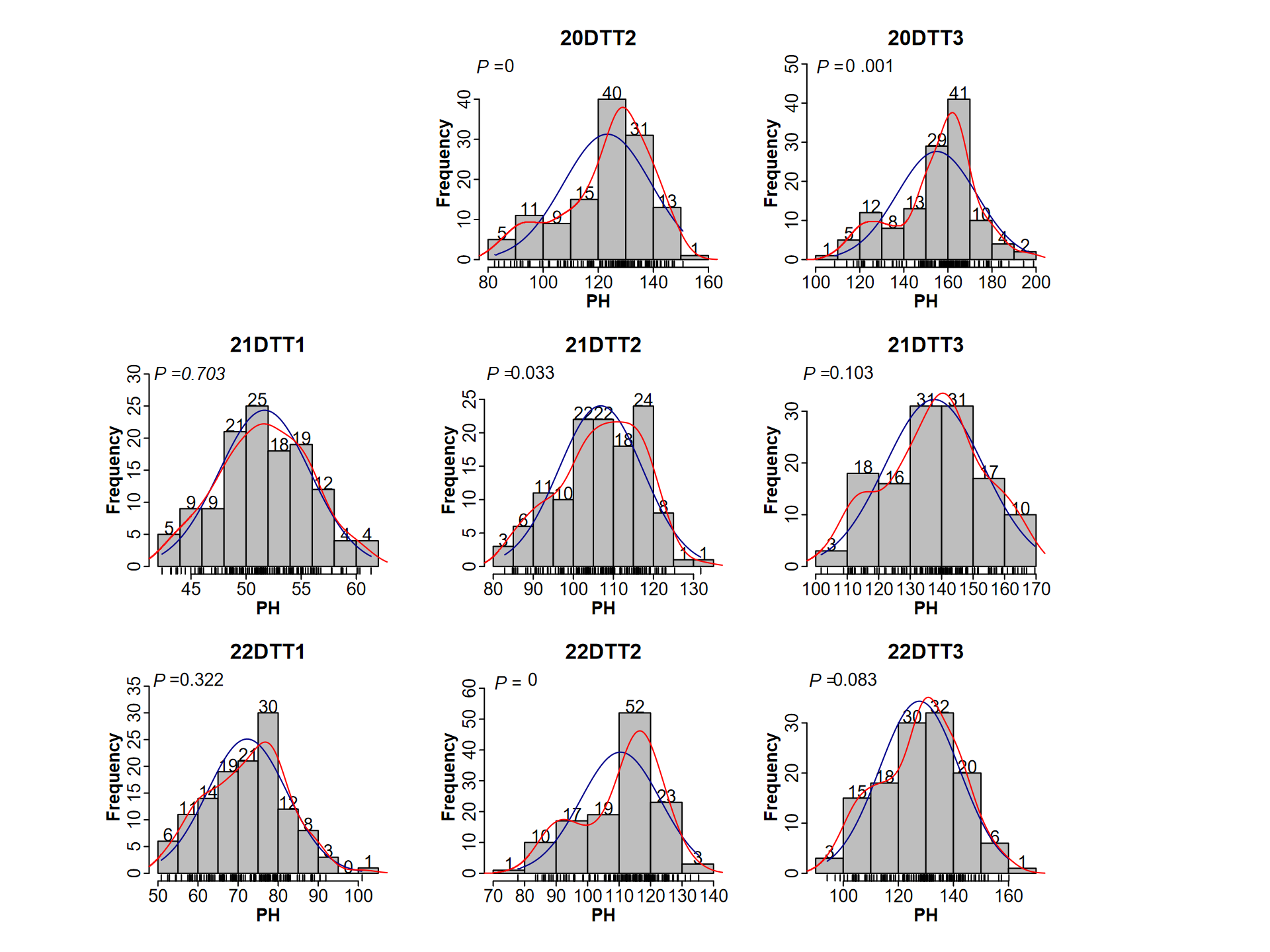

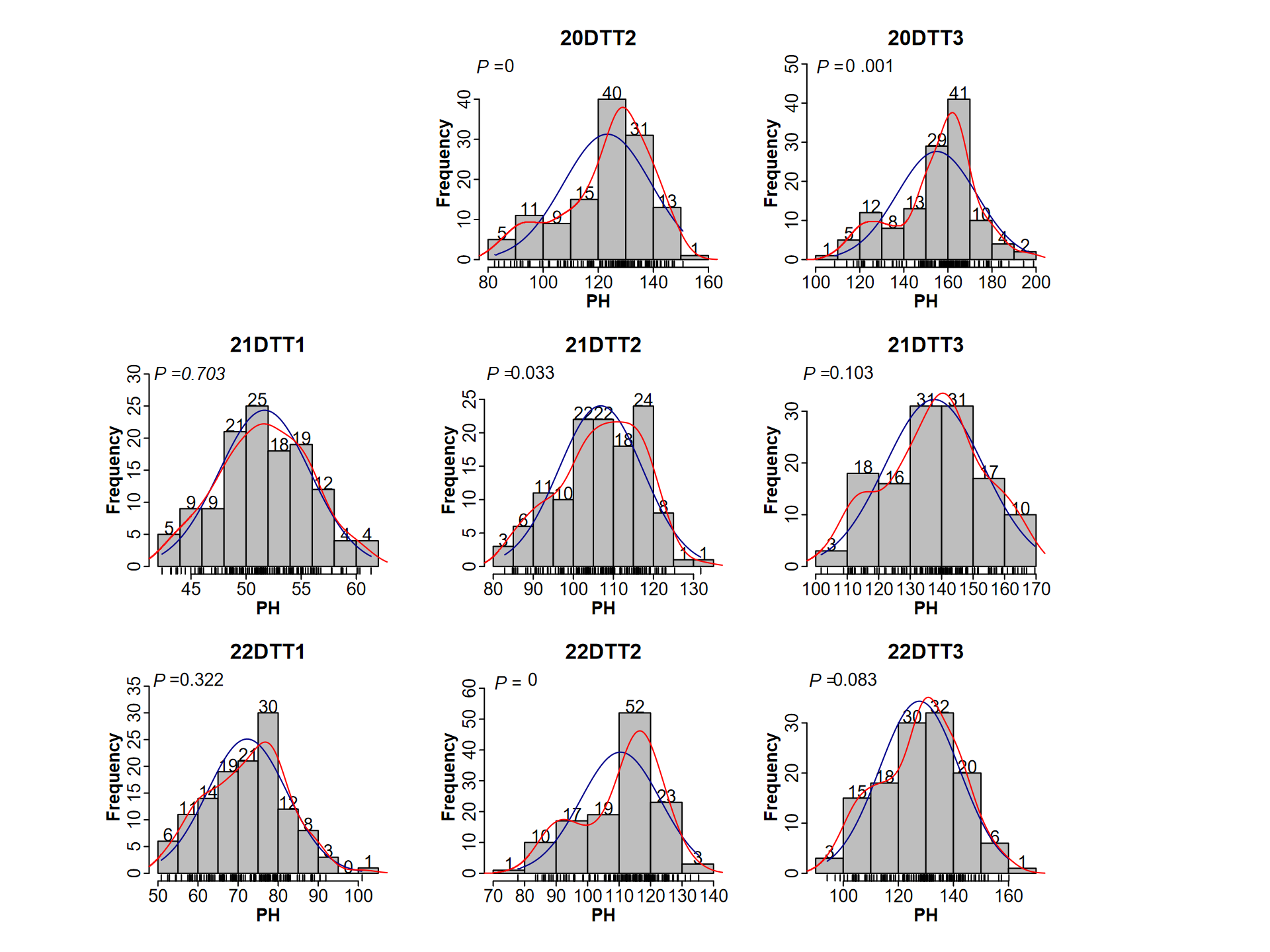

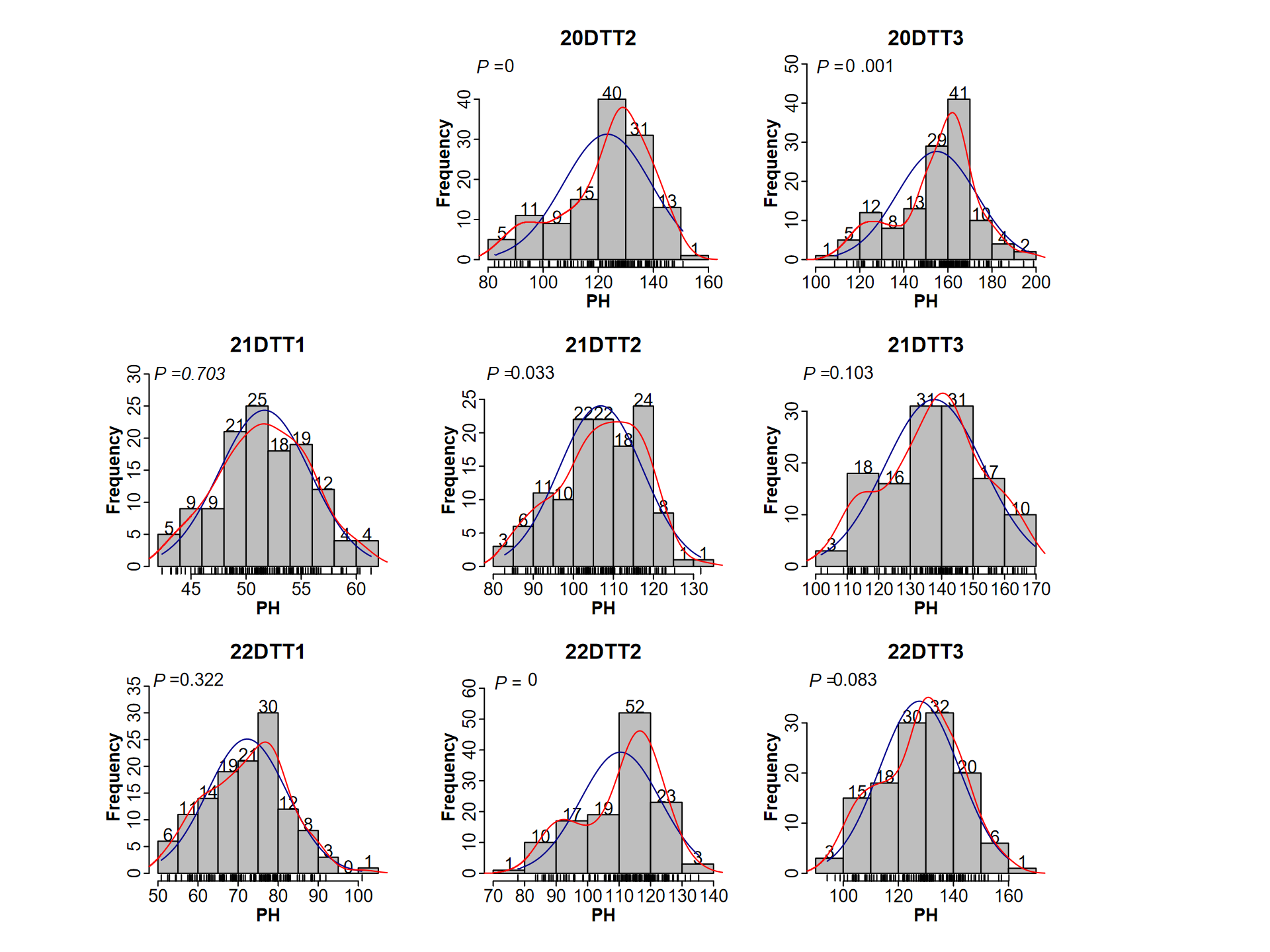


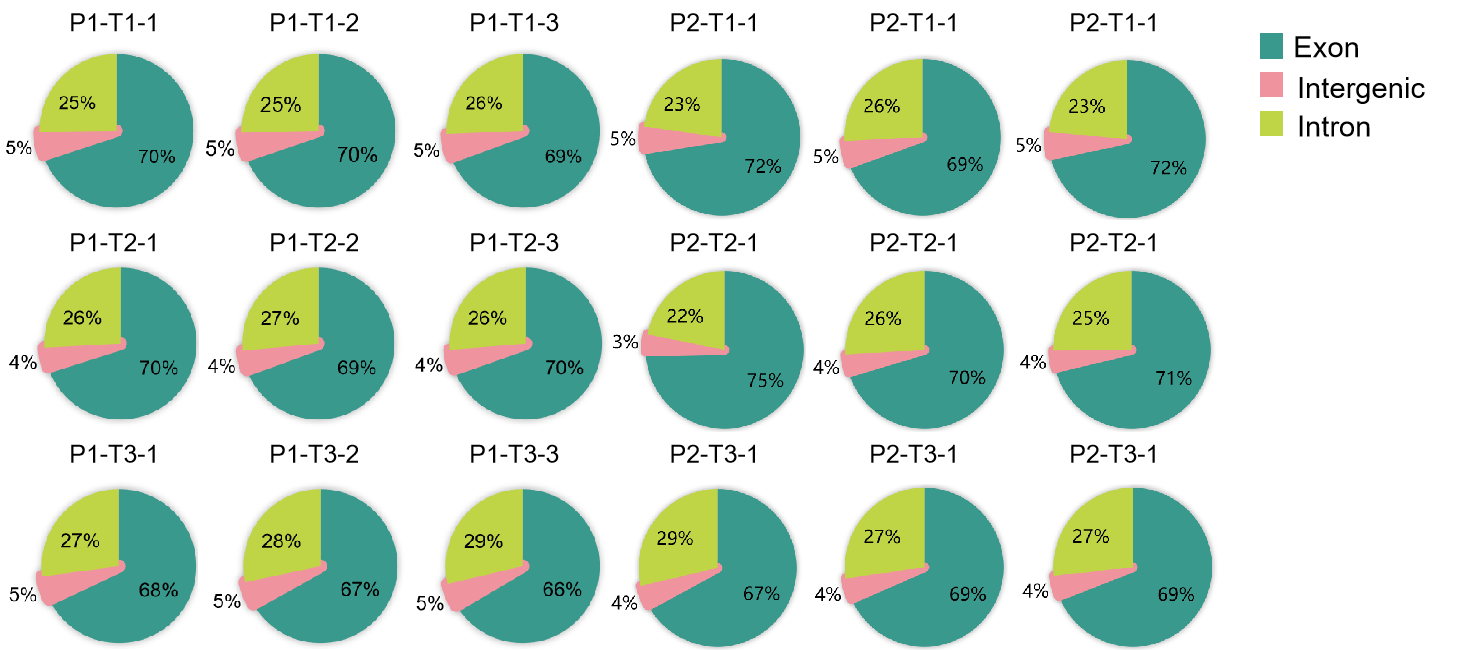


**Supplementary Figure S2** Reads Distribution across Different Genomic Regions of 18 samples. The genome is divided into exon regions, intergenic regions, and intron regions. The size of each region is determined by the percentage of reads mapped to that specific region out of all mapped reads.


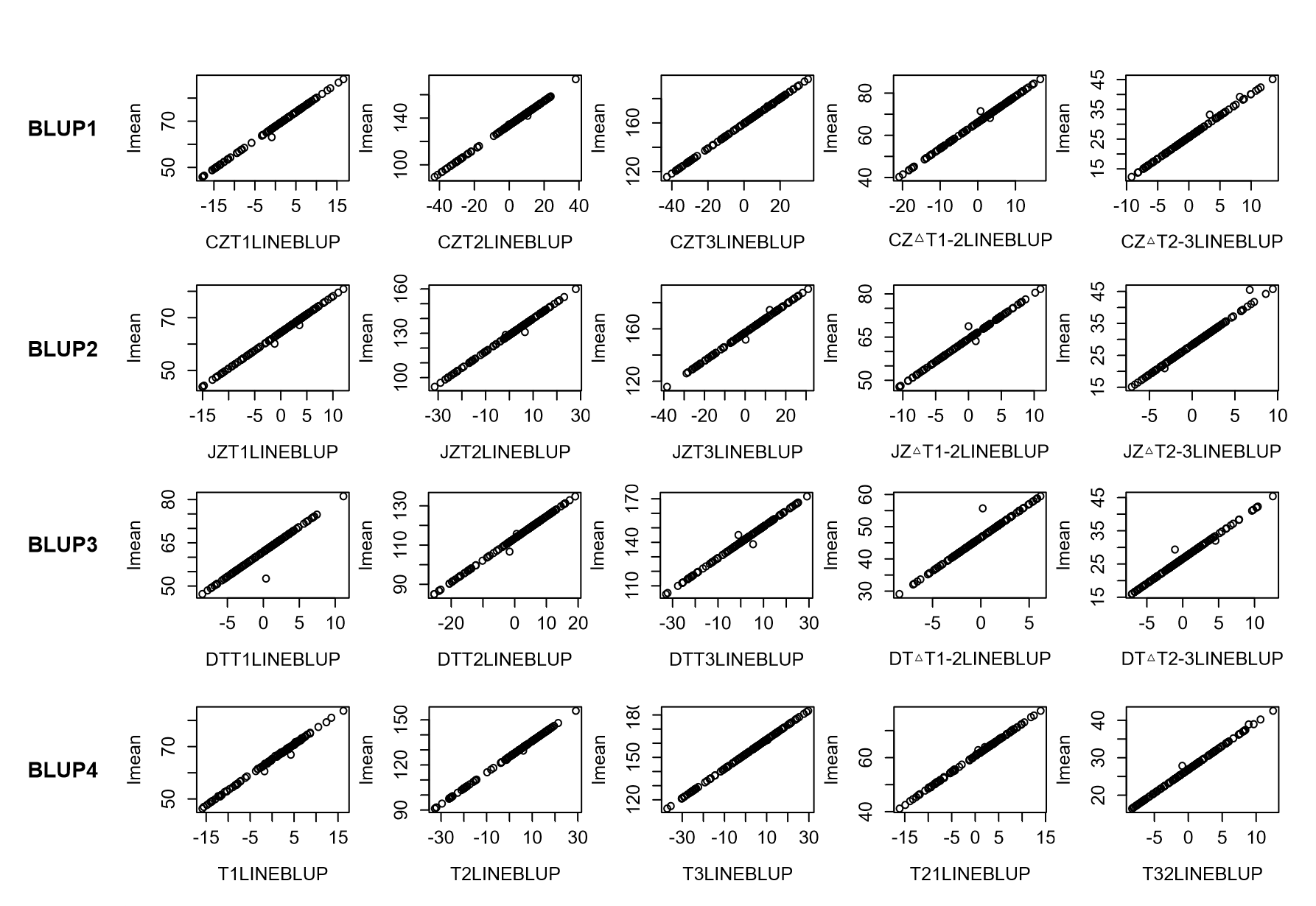
**Supplementary Figure S3** Mean of Best Linear Unbiased Predictions (BLUP) across Locations.
